# Evaluating the Impact of Washing Conditions on the Color Changes of Naturally Colored Cotton Fabrics: A Focus on Detergents, Water Types, and Temperature

**DOI:** 10.3390/ma17235777

**Published:** 2024-11-25

**Authors:** Hesam Aliei, Enric Carrera-Gallissa, Diana Cayuela

**Affiliations:** Institut d’Investigació Tèxtil i Cooperació Industrial de Terrassa (INTEXTER), Universitat Politècnica de Catalunya-BarcelonaTECH, 08222 Terrassa, Spain; hesam.aliei@upc.edu (H.A.); enric.carrera@upc.edu (E.C.-G.)

**Keywords:** natural-colored organic cotton, household washing, color changes

## Abstract

Intrinsically colored cotton is crucial for sustainability as it eliminates the need for chemical dyes, reducing water pollution and carbon footprint. It also preserves biodiversity by using fewer pesticides and supports eco-friendly, ethical product creation. This research aims to examine the factors that influence the color change that occurs in naturally colored organic cotton (NaCOC) fabrics when washed under normal household conditions, and it focuses on special detergents designed for people with skin hypersensitivity. The study observes the impact of various washing conditions on the color changes of the fabrics. Specifically, three specific detergents, two types of water (tap and distilled), and three different temperatures (20, 40, and 60 °C) are taken into consideration as variables. By using colorimetric measures and correlating the results with the significant variables of the experimental design, the study evaluates how washing practices affect both the color and the overall integrity of the fabric. The findings demonstrate that the water hardness is the most influential variable when it comes to the color changes in the fabrics. Additionally, higher washing temperatures exacerbate color changes, particularly in hard water conditions. These results provide valuable insights for maintaining the color integrity of NaCOC fabrics during washing.

## 1. Introduction

Cotton accounts for 22% of global textile fiber production and is the leading natural fiber in consumption due to its exceptional textile qualities and comfort [1]. In contemporary times, the growing concern for ecological issues has increased the fascination with naturally colored organic cotton (NaCOC). According to archaeological findings, this fiber has been used for at least 4500 years [2,3,4,5,6]. However, its applications in the industrial field remain relatively limited.

The extensive cultivation of NaCOC on a larger scale can be largely attributed to the advancements made by Sally Fox, a Californian entomologist. While working as an assistant to a producer [7], Fox first discovered wild brown cotton seeds and developed genetically resistant strains [8]. Although Fox was enthusiastic about colored cotton fibers, their poor textile quality posed a significant challenge for the spinning process. As a result, Fox focused on improving the textile properties of the fibers. Using careful selection and cross-pollination techniques, Fox developed colored cotton hybrids in 1988 that had fibers long enough to be spun using contemporary machinery. The success of cultivating naturally colored, spinnable cotton fibers eventually led Sally Fox to establish Natural Cotton Colors Inc. Additionally, she obtained a plant variety protection certificate for the cotton and registered it as FoxFiber.

The main appeal of NaCOC lies in its organic agricultural production, which eliminates the need for dyeing, thus avoiding the use of chemicals and water, resulting in a marked reduction of its environmental impact. The organic production of NaCOC contributes to agricultural sustainability by minimizing the use of pesticides and chemical fertilizers, promoting healthier practices for the environment and farmers. Furthermore, the absence of pesticides in the cultivation of the plant, along with the absence of chemicals associated with dyeing and finishing processes, makes garments derived from these fibers especially suitable for babies and people with hypersensitive skin and multiple chemical sensitivity (MCS). Determining the precise prevalence of MCS is challenging, as it varies significantly between different studies. Prevalence rates tend to be lower when patients undergo medical evaluation and can range from less than 1% to 33% in various studies conducted in Europe [9].

The brown color of NaCOC cotton is primarily attributed to proanthocyanidins and tannins, which are polyphenolic compounds that accumulate in the vacuoles of the fiber. These pigments develop towards the end of the plant’s growth cycle, particularly when the fibers are exposed to oxygen and sunlight. Genetic and environmental factors, such as climate and soil type, play a significant role in color variation, which may fade with prolonged exposure to sunlight. Furthermore, research indicates that catechins and tannin derivatives are essential components of these pigments, with their intensity potentially increasing or decreasing over time and with washing [10,11,12,13,14,15,16]. Grigorev et al. conducted a study analyzing the differences in metabolites of white and brown naturally colored cotton in relation to their implications for biofunctional and aseptic textiles. The findings on the properties of bioactive metabolites suggested therapeutic, delicate aseptic, repellent, UV protective, and metal toxicity-reducing effects of the studied fibers and their resistance to biodestruction by pecto- and cellulolytic bacteria and mold fungi. This would make biofunctional textiles more comfortable and hygienic [17].

To date, several studies have been conducted on the color changes experienced by fabrics made from NaCOC, focusing especially on the effects of different pre-treatments for industrial dyeing with chemical dyes (scouring, bleaching, etc.), as well as, though less extensively, on the effects of domestic washing. These studies have revealed that washing can substantially affect the visual characteristics of these textiles, particularly in lightness and saturation. Specifically, the scouring and washing processes have a considerable impact on the color and quality of NaCOC cotton. Unlike synthetically dyed cottons, the colors of NaCOC cotton can become more vibrant after several washes, reaching their peak intensity between 10 and 20 cycles for brown shades. Research also indicates that hard water and high temperatures exacerbate color variations. Alkaline and enzymatic treatments modify the brightness and chroma of the fibers, particularly in the case of green cotton, which affects its pigment. Conversely, washing additives such as optical brighteners and chlorine can lead to significant changes in colors, with chlorine having the most pronounced effect [7,8,18,19,20,21,22,23,24,25,26].

**Figure 1:**
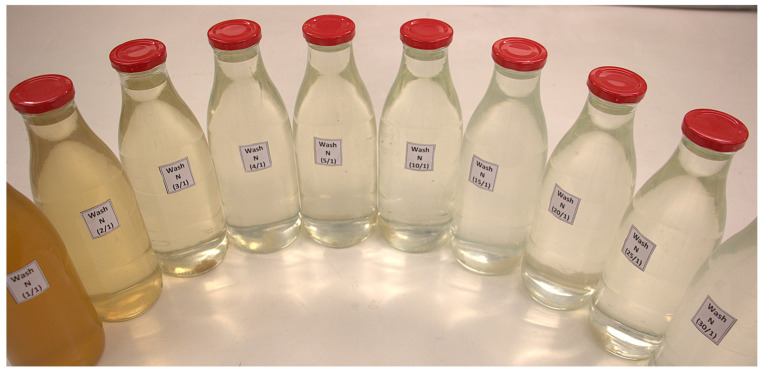
Color of the residual water from successive domestic washes.

In a previous paper [27], authors studied how household washing influences the colorimetric characteristics of NaCOC fabrics after undergoing washing for up to 30 cycles. Washing significantly changed the lightness and saturation of the samples, being the most significant difference in color between the two samples after the initial wash. Between the second and fifth washes, there are notable differences in these parameters. However, from the fifth wash, the variation in color difference was minimal. In this previous article, two distinct and opposing phenomena were observed regarding color change. First, there was fabric shrinkage, particularly after the first wash, because of the well-known relaxation shrinkage caused by the release of manufacturing tensions in cotton fabrics. This phenomenon increased the perceived color intensity of the substrates. On the other hand, a second phenomenon was observed, corresponding to the loss of pigments during successive washes, particularly in the first wash (Figure 1). In this case, it was determined that during the first wash, there was an extraction of waxes and oils from the cotton as well as a pigment loss of less than 1%. This phenomenon was associated with a decrease in color intensity. Since the overall result was an increase in color intensity, it was concluded that shrinkage was the dominant phenomenon influencing the change in the perceived color of the substrate.

The present study examines the color changes in NaCOC garments during the first five home washes, excluding industrial bleaching impacts, focusing exclusively on the changes that fabrics undergo during the usage phase of these garments by a broad group of users who have high skin sensitivity and therefore are required to use only specific commercial detergents for this condition. This study builds on previous [27] focusing on how different detergents, water types, and temperatures affect the colorfastness of NaCOC fabrics under household washing conditions, aiming to enhance their durability and consumer satisfaction. By evaluating how these specific detergents affect the characteristics of NaCOC, valuable information can be obtained on the durability and maintenance of these textiles. This, in turn, promotes more conscious and sustainable consumption, as garments that maintain their visual and functional properties for longer reduce the need for frequent replacement, thereby decreasing the environmental impact associated with textile production. Ultimately, this study contributes to a broader understanding of how sustainable textiles can be effectively integrated into consumers’ daily lives, fostering more ecological and responsible practices in the fashion industry.

In summary, because NaCOC cotton is characterized by being organic and containing fewer pesticides than the chemicals used in dyeing processes, it is an ideal fiber for babies and individuals with skin hypersensitivity issues. For this reason, the study is focused on the domestic washing behavior of this group using commercially available soaps that have a low environmental impact and are suitable for sensitive skin. Compared to those published to date, the novelty of this study is that it does not concentrate on industrial washes but rather on how domestic washing conditions with specific soaps produce changes in the properties of fabrics made from NaCOC cotton.

## 2. Materials and Methods

### 2.1. Material

This study employed naturally colored organic cotton from Brazil, supplied by the Spanish company Organic Cotton Colours (OCC), Santa Cristina d’Aro, Girona, Spain. The yarn used in this study was produced from a selection of OCC cotton varieties, including Topaz, Light Ruby, Dark Ruby, and Raw (white). The characteristics of these unique varieties, determined using high-volume instruments (USTER^®^ HVI 1000, USTER Technologies AG, Zurich, Switzerland), are listed in Table 1.

With these fibers, the OCC company made a mixture in equal proportions with the four cotton samples described in Table 1 and proceeded to carry out a combed spinning process (opening, cleaning, carding, drawing, combing, drawing, and finally, roving machine). Ring spinning was carried out on a PINTER machine model Merlin spa 1803, Pinter, Santpedor, Barcelona, Spain, under the conditions indicated in Table 2, which includes the characteristics of the yarn obtained.

The woven fabric used in this study was constructed using a conventional cotton warp (raw white) and a NaCOC weft produced on an air-jet Dornier loom combined with a Staübli Jacquard machine, Stäubli Española S.A.U., Sant Quirze del Vallès, Barcelona, Spain. The fabric exhibited a five-satin weave pattern so that the NaCOC weft threads emerge better on the surface of the fabric, the hems are distributed in a more regular way, and in this way, the color of the fabric is dominated by the high quantity of NaCOC weft yarns. The density is 38.4 warp yarns per centimeter (2/30 Nm), 19 weft picks per centimeter (2/34 Nm), and a total fabric width of 146 cm. The areal density is 314 g/m^2^. No other treatment has been applied to the fabric.

### 2.2. Domestic Wash

This study evaluated the color changes in domestic washing of three commercial detergents: Fox Fibre^®^ Colorganic^®^, Brooks, CA, USA (Det A [28]), Klar (Det B [29]) Almawin, Winterbach, Germany, and Pure Nature, Pure Nature, Idar-Oberstein, Germany (Det C [30]). All of them are designed for washing delicate textiles and are compounded to remove dirt and stains while safeguarding textile fibers effectively. These detergents have a minimal environmental impact. Table 3 includes data about these detergents.

The household wash was applied in a Fagor, model Innovation, Fagor Electrodoméstico, Madrid, Spain, to fabric samples measuring 30 × 30 cm. The tests were carried out following the concentration instructions provided by the producers for the three different detergents: 15 mL of Det A, 30 mL of Det B, and 30 mL of Det C. The substrates were washed five times (*X*_1_) using either distilled water or tap water (*X*_2_) at 20, 40, and 60 °C (*X*_3_). The hard water used corresponds to tap water from the city of Terrassa (Barcelona, Spain) and has a hardness between 360 and 400. mg CaCO_3_/L, depending on the day of the experiment. The pH of the detergent at the concentration used in household washing is 8.6, 8.7, and 9.0 for Det A, Det B, and Det C, respectively. The duration of the washing cycle varied with the temperature: 50 min at 20 °C, 60 min at 40 °C, and 80 min at 60 °C.

After washing, the fabric specimens were dried using a Rommer Dry 92 tumble dryer, Terrassa, Spain. Subsequently, they were conditioned in a standardized environment maintained at 20 °C and 60% relative humidity for 24 h.

### 2.3. Characterization

Color changes were determined by colorimetry using a GretagMacbeth Color i7, X-Rite Ibérica, L’Hospitalet de Llobregat, Spain, instrument conforming to the ISO 105-J03 standard [31] with measurements taken under a D65 illuminant and a 10-degree observer angle. The degree of color change between each laundered fabric sample and its original state was determined using the color difference parameter, ∆E_ab_* (Equation (1)).

(1) $${{∆E}_{ab}}^{*}=\sqrt{{\left(∆L*\right)}^{2}+{\left(∆a*\right)}^{2}+{\left(∆b*\right)}^{2}}$$

where L*, a*, and b* are the lightness, green–red coordinate, and blue-yellow coordinates, respectively, in the CIEL*a*b* space.

The Chroma of the samples is described trough the C*_ab_ value that is related with a* and b* in the CIEL*a*b* color space (Equation (2)):

(2) $${C^{*}}_{ab}=\sqrt{a^{2}+b^{2}}$$

The hue (*h*) is also related to a* and b* with Equation (3).

(3) $$h_{ab}=arctg\left(\frac{b}{a}\right)$$

The shrinkage of the fabrics after laundering was determined according to ISO 3759:201 [32].

### 2.4. Modeling

The color difference response was analyzed by modeling with quantitative and qualitative variables, as indicated in Table 4.

Variations of the different parameters according to washing conditions were modeled by multiple linear regression. To better analyze the influence of each parameter, coded factors were used according to Equations (4)–(6) for the number of washes (*N*, *X*_1_), type of water (*W*, *X*_2_), and test temperature (*T*, *X*_3_), respectively.

(4) $$X_{1_{i}}=\frac{N_{i}-\bar{N}}{∆N}$$

(5) $$X_{2_{i}}=\frac{W_{i}-\bar{W}}{∆W/2}$$

(6) $$X_{3_{i}}=\frac{T_{i}-\bar{T}}{∆T}$$

where $\bar{N}$, $\bar{W,}$ and $\bar{T}$ are the mean of the factors levels, and ∆N, ∆W, and ∆T are the differences between two consecutive levels for each factor.

Categorical variables were used to introduce the detergent variable (qualitative) into the model, taking on the values specified in Table 5 for each level (Det A, Det B, Det C).

When all categorical variables have a value of zero, the model for detergent A will be estimated. This detergent will be the reference, and the model will indicate if there are significant differences between the two types of detergent. If, in the final model, there are terms involving *Q*_2_ and/or *Q*_3_, it will indicate that there are statistically significant differences for the different types of detergents. By substituting the values of *Q*_2_ and *Q*_3_, separate models can be obtained for the different types of detergents.

The influence of the factors on each response, *Y*, was analyzed through regression analysis, fitting the empirical model shown in Equation (7).

(7) $$Y=\beta_{o}+\sum_{i}\beta_{i}X_{i}+\sum_{ij}\beta_{ij}X_{ij}+\varepsilon \;i,j=1,2,...;i\geq j$$

where $\beta_{o}$, $\beta_{i}$, $and\;\beta_{ij}$ are the coefficients to be estimated with regression analysis, and ε is the disturbance term (noise). The significance of the model was assessed by the analysis of variance [33], and the non-significant coefficients (95% confidence level) were removed from the model to obtain the “best” regression equation [34].

## 3. Results

Firstly, the fabric was analyzed to determine whether any microstructural changes had occurred during washing. To identify any potential relative changes or deformations in the characteristic bands of cotton due to microstructural changes, the ATR (attenuated total reflectance) spectrum of the fabric was analyzed. Given that the surface concentration of waxes and pigments is significantly lower than the approximately 5% threshold required for ATR detection, any observable changes would likely result from microstructural alterations. However, the results obtained (Figure S1 in the Supplementary Information), which compare the spectra of the original fabric with those washed 1 and 30 times, show no differences between them after ATR analysis. This suggests that no microstructural differences were detected in the various substrates after washing.

In the previously cited study, where one detergent, one temperature, and one type of water were examined, shrinkage during the first wash was identified as the factor that most influenced color change. During mechanical and chemical processing, fibers, yarns, and fabrics are subject to mechanical tensions. In addition, some textile structures are not balanced from the mechanical point of view (a typical example is the twill weave in denim fabrics), increasing residual internal stresses in the fabric. Since the numerous small or larger spaces between fibers and yarns give them the freedom to move, mechanical actions occurring during laundry (in combination with water acting as a lubricant) may allow the relaxation of internal stresses, leading to visible shape changes in the clothes [35]. Therefore, shrinkage was the first parameter to be analyzed in the experimental design. The analysis showed that shrinkage was independent of the type of water and detergent, and it changed very little with the number of washes. In fact, the average shrinkage in the first wash of all substrates (considering all temperatures, detergents, and types of water) was 6.8 ± 0.7%, and the average shrinkage across all washes was 7.6 ± 0.7%. Based on these results, the studied variables (number of washes, temperature, and type of detergent) did not have a significant impact on the fabric’s shrinkage, and, therefore, the differences among the substrates obtained after washing in the different conditions must be attributed to the significant variables of the experimental design.

The reflectance of the original substrates and the substrates washed with all specified detergents, using both distilled and tap water at different temperatures, is shown in Figures S2–S4 of the Supplementary Information for the tests at 20, 40, and 60 °C, respectively. The most significant changes in reflectance were primarily observed during the first washing cycle, where a decrease in reflectance occurred for all substrates. According to the curves, the reflectance curve decreases when the washing is carried out in hard water. The CIEL*a*b* coordinate values derived from the reflectance measurements were studied for a more in-depth analysis.

Results for washing with detergents A, B, and C are included in Tables S1–S3 of the Supplementary Information, respectively. From the results, there were alterations in fabric color when the number of washes was increased under different washing conditions.

**Figure 2:**
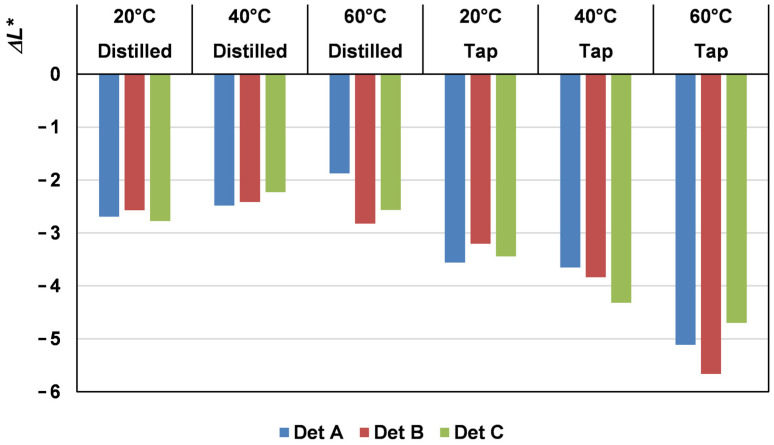
ΔL* of all the samples after the first wash.

The lightness, L*, decreases in an important way after the first wash, indicating noticeable darkening. The change in lightness, ΔL*, in this first wash for the samples is included in Figure 2.

In all cases, except for detergent A washed with distilled water at 60 °C where ΔL* was −1.9, the decrease was greater than two units. Considering that a color difference between the two units was noticeable to the naked eye if only the change in luminosity was taken into account in Equation (1), the error would be at least two units. This implies that the first wash results in a darkening of the sample that was visible to the naked eye. No significant differences were observed between the various detergents and temperatures when washing with distilled water, but the differences at different temperatures were important when washing with hard water.

After this first wash, although a decrease of ΔL* existed, the average difference between the second wash and the fifth was 0.65. No significant differences among the different conditions existed.

In order to identify accurately the variable or variables that had the greatest impact on the color change in the first domestic wash, the Equation (7) model was employed. As only the first was studied, the X_1_ variable was not included, being the studied answer the ΔL* of the first wash (Equation (3)).

The resulting model of ΔL* of the first wash is as follows:

ΔL* = −3.329 
− 0.837·*X*_2_ − 0.223·*X*_3_ − 0.504·*X*_2_·*X*_3_  − 0.456·*Q*_3_·*X*_3_ R^2^ = 0.945 (8)

In the model, it was observed that both variables *X*_2_ (type of water) and *X*_3_ (temperature) had a significant impact on the luminosity response ΔL* of the first wash, along with their interaction. The type of water interacts with the temperature, and the interaction of detergent B with temperature was significant. The coefficient of determination for the fit was 94.45%, indicating a strong fit of the estimated model to the experimental data.

Upon substituting the values of the categorical variable *Q*_3_, the following models were derived (Equations (9) and (10)):

Det A and Det C   ΔL* = −3.329 − 0.837·*X*_2_ 
− 0.223·− 0.504·*X*_2_·*X*_3_ (9)

Det B   ΔL* = −3.329 − 0.837·*X*_2_ 
− 0.679·*X*_3_ − 0.504·*X*_2_·*X*_3_ (10)

The results indicated that, apart from the shrinkage as a main factor, water hardness was the primary factor affecting the decrease in luminosity in the initial wash of NaCOC fabrics. The higher the water hardness, the more pronounced the darkening effect. Additionally, although to a lesser extent, temperature also played a significant role, particularly in the case of detergent B. When using distilled water, the darkening between the washed fabrics and the original one was reduced. However, this difference became more significant as the water hardness increased, especially at higher temperatures. Considering that luminosity ranges from 0 for black to 100 for white, the negative sign of all the coefficients in the model indicated that the higher the values of water hardness and temperature, the lower the luminosity. In other words, a darker color was perceived.

In order to properly analyze the chroma and hue, a prior understanding of the pigments responsible for color is necessary. The brown color in naturally colored cotton fibers was primarily due to the presence of tannin pigments, as extensively studied by Ma et al. [14,15] and by Pen et al. [16].

Tannins can vary in their solubility in water depending on their chemical structure and molecular weight. Generally, smaller tannin molecules were more soluble in water, while larger tannin molecules tended to be less soluble. The solubility of tannins in water also depends on factors such as pH, temperature, and the presence of other solutes.

Then, in the case of naturally colored cotton, some of these tannins present in the fibers may be soluble and contribute to the color during processing; others may be tightly bound to the cotton fibers and less likely to leach out during washing or other treatments.

**Figure 3:**
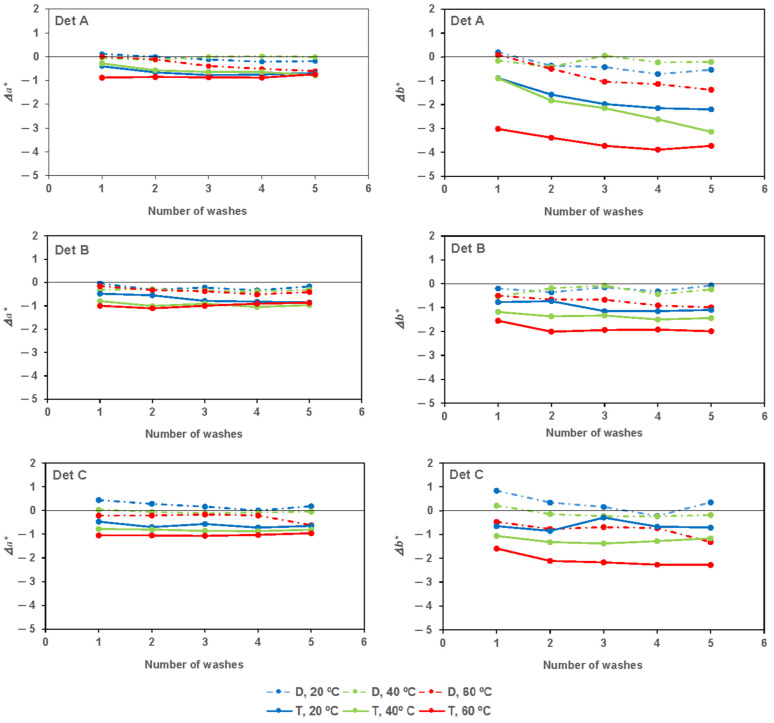
Δa* (**left**) and Δb* (**right**) for detergents under the different washing conditions, unwashed fabric being the reference.

Figure 3 represents Δa* for detergents under the different washing conditions, unwashed fabric being the reference. In the first wash, there was a general decrease in the value of the green-red axis, a*, which was more pronounced when washed with hard water than with distilled water. Additionally, the figures showed a tendency for this decrease to be greater as the temperature of the domestic wash increases. On the other hand, there were no significant differences when increasing the number of washes. In other words, this value was significantly changed with the first wash but did not significantly change further with subsequent washes.

The yellow–blue axis, b*, also experienced a general decrease in its value in the first wash, which was more pronounced when washed with hard water than with distilled water. Furthermore, it can be observed in the figures that there is a tendency for this decrease to be greater as the temperature of the domestic wash increases. On the other hand, it seemed that in some cases, especially when washing with detergent A, there was a certain decrease in this value as the number of washes increased. In other words, generally, there was a significant change in this axis with the first wash, but it no longer changed or changed very little with subsequent washes.

The decrease in both a* (in the positive semi-axis, corresponding to red) and b* (in the positive semi-axis, corresponding to yellow) indicated a decrease in the colors red and yellow (components of brown).

**Figure 4:**
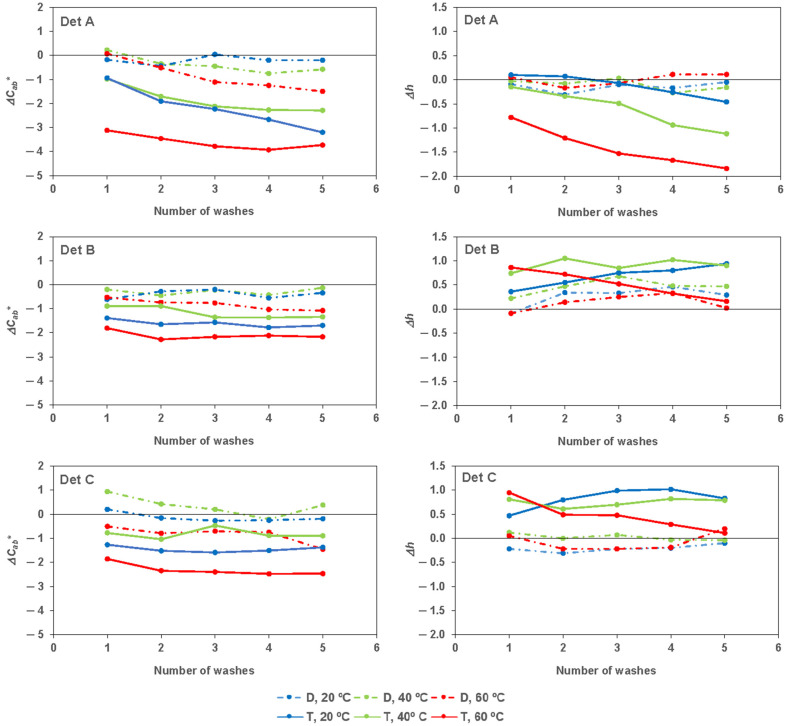
ΔC*_ab_ (**left**) and Δ*h* (**right**) for detergents under the different washing conditions, unwashed fabric being the reference.

Figure 4 represents ΔC*_ab_ and Δ*h_ab_* for all the detergents under the different washing conditions. The decrease in chroma values (ΔC*_ab_) followed a similar order and trend as those of b*, given that the decrease in a* was much smaller than that of b*, resulting in the chroma trend being similar to that of b*.

In general, a decrease in chroma in a fabric after washing refers to a reduction in the intensity or purity of its color. When fabric undergoes washing, especially if it involves harsh detergents, agitation, or exposure to sunlight, the color molecules can break down or fade. This can result in a decrease in chroma, making the color appear duller or less vibrant. Factors such as the type of fabric, dye used, washing method, and water temperature can all affect the degree of chroma loss.

As studied by Ma [14,15], the tannin concentration and/or type of tannin were related to the hue of the fabric. The hue, pure color of an object, remained virtually unchanged or slightly decreased when washing at higher temperatures in tap water with detergent A. For detergents B and C, it either remains unchanged or slightly increases in the first wash and then remains constant. As the differences were minimal (Δ*h* approaches zero), the hue or tone between the samples became nearly identical. This indicates that, although there may be variations in other color attributes (such as brightness or chroma), the fundamental tone—whether it is red, blue, or green—remained almost unchanged among the samples.

A significant model relating the individual responses Δa*, Δb*, ΔC*_ab_, and Δ*h_ab_* for all the detergents to the process variables were obtained. The color difference is a global parameter that measures the variation in color and is more representative of the overall phenomenology. As for the color difference, ΔE*_ab_, ranged from a minimum of 2.5 to a maximum of 7.6. These values were well above a maximum accepted error value of two units.

**Figure 5:**
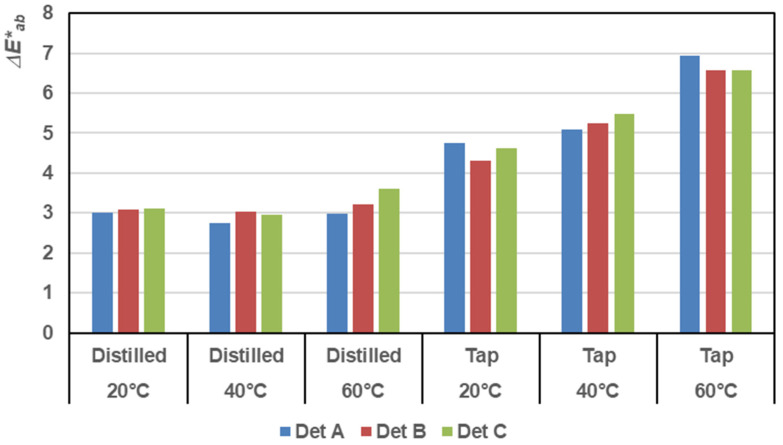
Comparison of ΔE*_ab_ values after five washes between washed and original samples with different detergents, water hardness, and washing temperature.

ΔE*_ab_ values increased with temperature, particularly with tap water, demonstrating a substantial color change with each wash. This emphasizes the impact of tap water at elevated temperatures on hastening color alteration. Figure 5 illustrates the color difference for all samples under various conditions to analyze the color change more thoroughly after multiple washes. No significant differences were observed when washing with distilled water and when using different detergents at different temperatures. However, the most notable differences were found when washing with hard water, where the temperature also played an important role.

In order to identify accurately the variable or variables that have the greatest impact on the color change of substrates obtained after a domestic wash, the Equation (7) model was employed. Interactions between the number of washes and temperature (*X*_1_·*X*_2_) or type of water (*X*_1_·*X*_3_) were not considered. The beta coefficients were estimated from this initial model using the linear stepwise backward regression model with a significance level (α) of 0.05.

The resulting model of ΔE*_ab_ is as follows (Equation (11)):

ΔE*_ab_ = 4.266 + 0.304·*X*_1_ 
+ 1.379·*X*_2_ + 0.518·*X*_3_ + 0.456·*X*_2_·*X*_3_ 
− 0.236·*Q*_2_·*X*_2_ − 0.262·*Q*_3_·*X*_2_ R^2^ = 0.949 (11)

In the model, it was observed that the variables *X*_1_ (number of washes), *X*_2_ (type of water), and *X*_3_ (temperature) were significant in the response ΔE*_ab_. The type of water interacts with the temperature. In the equation, there were significant differences in the qualitative variables because there were interactions of terms in Q with water hardness that were different from zero. The coefficient of determination of the fit was 94.87%, a very high value that indicates a good fit of the estimated model to the experimental data.

It was observed that the highest coefficient obtained was for *X*_2_ (type of water) and that this variable interacts with all the process variables, except for the number of washes and interaction previously disregarded (due to previous individual analyses with each detergent).

By substituting the values of the categorical variables for each type of detergent, the models shown in Table 6 are obtained.

The obtained results show that water hardness is the variable that most influences the color change of NaCOC fabrics, so the higher the water hardness, the greater the color change. Additionally, this variable depends on the type of detergent, although the differences between detergents are not very significant (coefficients between 1.12 and 1.38). The hardness of the water from three solutions prepared with the same concentrations as those of the detergents found in household laundry was analyzed to delve deeper into the topic. The results showed a 240, 250, and 210 mg CaCO_3_/L hardness for detergents A, B, and C, respectively. From the results, detergents exhibit a complexing ability for calcium and magnesium cations found in water that is quite comparable, owing to the presence of the sequestrants incorporated within them. It was also observed that the detergents that exhibit the highest and lowest coefficients in *X*_2_ (1.379 and 1.117) demonstrated very similar water hardness levels (240 and 250 ppm, respectively). Then, the hardness of water was indeed associated with color change; however, the reason for this was not solely due to the varying levels of chelating agents in its compositions.

In the evolution of pigment colors during washing, the desorption of the pigment from the fabric is one phenomenon that must be considered, that is typically associated with the solubility of the pigments used, as well as the stabilization of species in the medium due to the action of surfactants. The effectiveness of surfactants was determined by their ability to emulsify the pigment molecules that were released from the fabric into the wash water. This effect varies among different pigments and surfactants, depending on the organic nature of the pigment (log P) and the surfactant’s hydrophilic–lipophilic balance (HLB). Depending on the characteristics of the surfactant, which may involve mixtures, the stabilization of the pigment in the wash water will vary. The greater the stabilization, the more pigment will be released, resulting in a change in the color of the fabric’s surface.

Among the surfactants used, Det A contains an anionic surfactant and several emulsifying non-ionic surfactants, which better stabilize the pigment. It will form a mixed micelle (anionic-non-ionic) that is highly stable. Det B is a soap that will only marginally form stable micelles with the dye. Its effect on reducing interfacial tension and emulsification is less significant compared to the other detergents. Det C primarily consists of non-ionic surfactants with a small amount of lauryl sulfate. It will create a mixed micelle, but it will be less stable due to the predominance of non-ionic surfactants.

A second phenomenon that could explain the color differences is the ingress of ions present in the water, which is related to water hardness; thus, the higher the hardness, the greater the influx of ions. The presence of ions in absorbent substrates, such as the studied NaCOC cotton fabrics, can alter the color through the complexation of the pigment by surfactant molecules (particularly those containing SO_3_^−^) that penetrate into the fabric.

The resonance exerted by the surfactant on the pigments led to a variation in the pigment’s shade. This phenomenon was noted in the changes of parameters a* and b*. A decrease in parameter a* occurred during the first and/or second wash, after which it remained constant. Conversely, the value of parameter b* decreased as the number of washes increased when the wash was carried out in the presence of Det A, which contains SO_3_^−^, even up to the 30 washes examined in a previous study [27]. This was attributed to the increasing amount of surfactant that binds to the surface of the pigments as the fabric was washed. This effect was further confirmed by the reduction in the chroma, C*_ab_, of the substrates with successive washes and, consequently, the value of the color difference (ΔE*_ab_) in the fabrics.

Next, the washing temperature variable followed with an important role, which had a significant interaction with water hardness. The lower the water hardness (distilled water), the smaller the color difference between the washed fabrics and the original, and this difference increased significantly with temperature as water hardness increased.

The number of washes had less importance (coefficient = 0.304), so the color difference depended little on this value.

Finally, it is very important to indicate that only the weft yarn was NaCOC cotton. In the case that both the weft and warp threads were NaCOC, the color difference would be much more significant.

## 4. Conclusions

This study examines the impact of domestic washing on the color variation of naturally dyed cotton fabrics. The variables analyzed include the number of washes, the type of water used, and the temperature, with the responses being shrinkage and color parameters, primarily lightness (L*) and color difference (ΔE*_ab_). The textiles were laundered using detergents specifically formulated for individuals with skin hypersensitivity and that are environmentally friendly.

According to a previous study, shrinkage is the variable that most significantly affects the initial color change. This research has shown that, in addition to shrinkage, among all the factors considered, the type of water is the most influential variable in the overall color alteration of the fabric, with the most significant change occurring after the first wash. Notably, tap water has caused more pronounced changes than distilled water in terms of both lightness and color difference. Color alteration occurs through the absorption of surfactants into the fabric, aided by calcium and magnesium, which reduce the negative charge on the fabric’s surface, facilitating this absorption. These surfactants can affect light absorption by the pigment due to their interaction with the pigments, acting as auxochromes and modifying light absorption. This finding suggests that using water with lower hardness may be advantageous for maintaining fabric quality. Furthermore, the use of hard water necessitates larger amounts of detergent and additives to counteract the hardness, potentially leading to a greater environmental impact.

Temperature plays a crucial role as higher temperatures increase color change. This is because elevated temperatures facilitate the penetration of detergents into the fabrics, thereby enhancing the previously mentioned effect. Since washing at high temperatures affects the color change of fabrics, it is advisable to wash at lower temperatures to prevent darkening and alterations in color. Additionally, washing at lower temperatures is generally more energy-efficient, reducing electricity consumption and greenhouse gas emissions associated with water heating. Although the number of washes, starting from the first wash, contributes to a gradual decline in color effectiveness and intensity, its impact is relatively minor.

## Figures and Tables

**Table 1:** Characteristics of the NaCOC fibers used.

Sample	MIC	MAT	UHML (mm)	UL (%)	SF (%)	STR (g/tex)	ELG (%)
Topaz	4.23	0.84	26.67	79.5	9.5	25.0	8.6
Light ruby	4.26	0.84	20.50	78.8	11.9	19.6	9.9
Dark ruby	4.15	0.84	21.01	79.2	12.0	21.7	9.0
Raw	3.76	0.84	25.73	78.0	10.3	26.3	7.0

where: MIC: Micronaire index. The pressure drop was measured by passing air through the fibers. MAT: maturity index. This indicates the degree of cell wall thickness in the cotton samples. The maturity index determined through HVI correlates very well with the AFIS maturity ratio and microscopy reference method (cross-sectional analysis). UHML: upper-half mean length. It is the mean length by the number of fibers corresponding to the class’s staple length and the AFIS upper quartile length by weight. UL%: uniformity index. It expresses the ratio of the mean length to the upper-half mean length. This indicates the distribution of the fiber length within the fibrogram. SF%: short fiber index. Indicates the percentage of fibers less than 12.7 mm in length. This correlates well with the AFIS short-fiber content by weight (SFC). STR: bundle strength is the breaking strength of the cotton fibers in grams per tex. ELG: elongation. This is a measurement of the increase in the length of the cotton test piece, expressed in %, as a result of the application of a load.

**Table 2:** Spinning conditions and characteristics of the yarn obtained.

Parameter	Spinning Conditions and Characteristics of the Yarn
Roving count (ktex)	0.600
Yarn count (tex)	29.5
Twist (t/m)	705
Linear spinning speed	16.65 m/min
Revolutions per minute of the spindle	7500 rpm
Ring diameter	42 mm
Type of traveller	Bräcker 2DR

**Table 3:** Information about the composition of the detergents.

Reference	Composition
Det A	Citrus Grandis fruit extract. Ammonium lauryl sulfate emulsifier. Cetyl alcohol EO. Soyamide DEA. Sodium oleate. Other ingredients: hydrolyzed cotton protein, sodium benzoate, potassium sorbate, citric acid, curcuma zedoaria, and CL 16035.
Det B	15–30% soap (soap and vegetable soap *). <5% non-ionic surfactants (carbohydrate surfactants). Other ingredients: water, ethanol, sodium citrate, lactic acid, and citric acid. 100% of the total ingredients are of natural origin. * From controlled organic farming.
Det C	15–30% anionic surfactants. 5–15% non-ionic surfactants. 5–15% plant alcohol. Other ingredients: water, potassium soap of rapeseed, oil (organic), sodium lauryl sulphate, alcohol denatured, lauryl glucoside, and octyl sulphate.

**Table 4:** Experimental plan variables and levels.

Type of Variable	Code	Factor	Levels Value	Coded Factor
Quantitative	*X*_1_	Washes number (*N*) (5 levels)	1	−2
			2	−1
			3	0
			4	1
			5	2
	*X*_2_	Type of water (*W*) (2 levels)	Distilled (0 ppm hardness)	−1
			Tap (400 ppm hardness)	1
	*X*_3_	Temperature (*T*) (3 levels)	20	−1
			40	0
			60	1
Qualitative		Type of detergent	Det A Det B Det C	See Table 5

**Table 5:** Categorical variables for each level.

	Level		
Categorical Variable	Det A	Det B	Det C
*Q*_2_	0	0	1
*Q*_3_	0	1	0

**Table 6:** Model for each type of detergent (*X*_1_ = number of washes; *X*_2_ = type of water; *X*_3_ = test temperature).

Level	Model
Det A	ΔE*_ab_ = 4.266 + 0.304·*X*_1_ + 1.379·*X*_2_ + 0.518·*X*_3_ + 0.456·*X*_2_·*X*_3_
Det B	ΔE*_ab_ = 4.266 + 0.304·*X*_1_ + 1.117·*X*_2_ + 0.518·*X*_3_ + 0.456·*X*_2_·*X*_3_
Det C	ΔE*_ab_ = 4.266 + 0.304·*X*_1_ + 1.143·*X*_2_ + 0.518·*X*_3_ + 0.456·*X*_2_·*X*_3_

## Data Availability

The original contributions presented in the study are included in the article/Supplementary Material, further inquiries can be directed to the corresponding author.

## Supplementary Materials

The following supporting information can be downloaded at: https://www.mdpi.com/article/10.3390/ma17235777/s1, Figure S1: Detail of the ATR between 4000 and 2100 cm^−1^ (up) and between 2100 and 600 cm^−1^ (down) of the original fabric and the 1 and 30 times washed; Figure S2: Reflectance curves of the substrates washed with detergent A; Figure S3: Reflectance curves of the substrates washed with detergent B; Figure S4: Reflectance curves of the substrates washed with detergent C; Table S1: CIE*L***a***b** coordinate values before and after washing with detergent A under different conditions; Table S2: CIE*L***a***b** coordinate values before and after washing with detergent B under different conditions.; Table S3: CIE*L***a***b** coordinate values before and after washing with detergent C under different conditions.
