# Supplementary material for: Evaluating the Impact of Washing Conditions on the Color Changes of Naturally Colored Cotton Fabrics: A Focus on Detergents, Water Types, and Temperature
Source: Materials (Basel). 2024 Nov 25;17(23):5777. doi: 10.3390/ma17235777 (PMC11642346; doi:10.3390/ma17235777)
Supplement: Supplementary file 1 [file materials-17-05777-s001.zip › materials-3283216-supplementary.pdf]

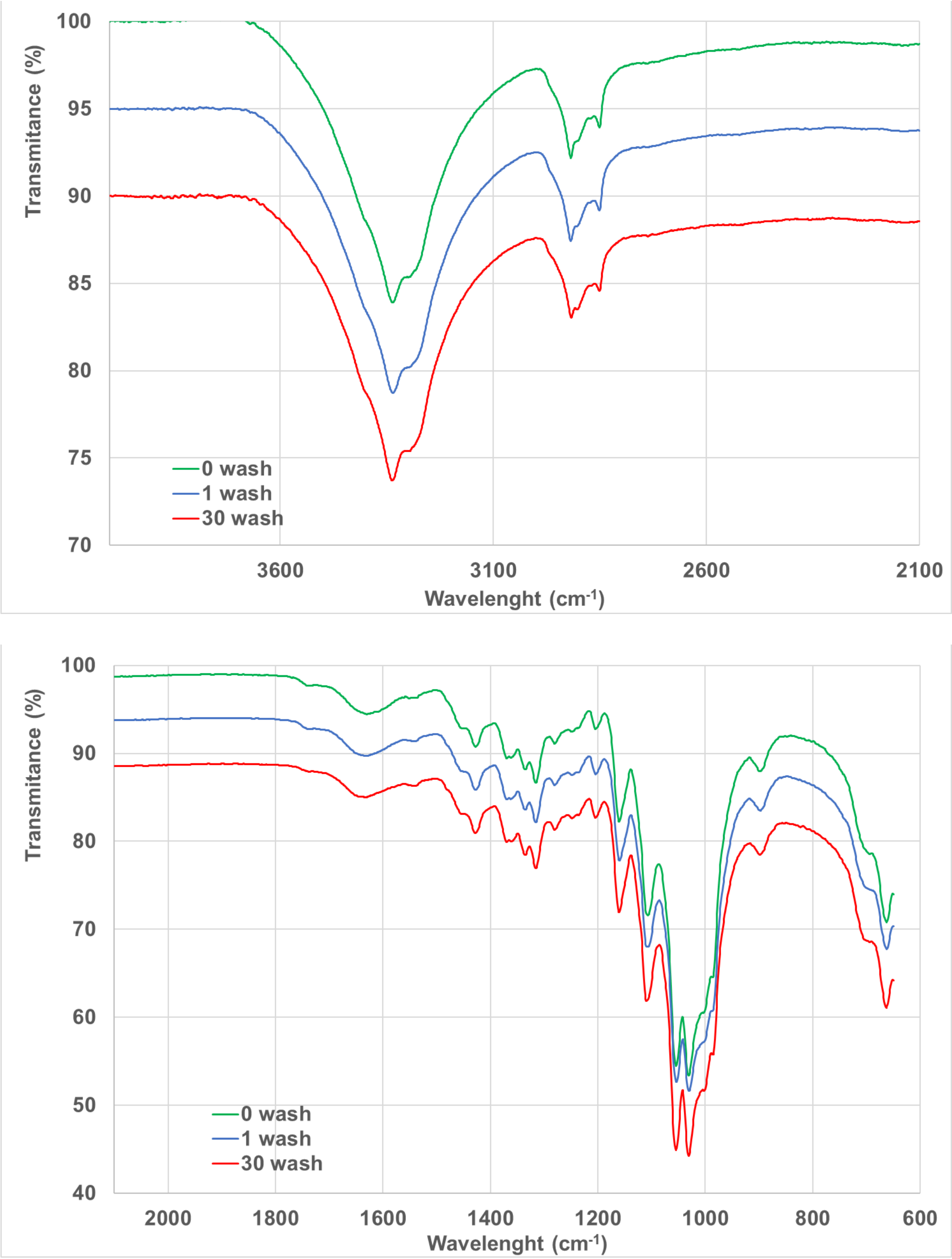

Figure S1. Detail of the ATR between 4000 and 2100 cm^{-1} (up) and between 2100 and 600 cm^{-1} (down) of the original fabric and the 1 and 30 times washed.

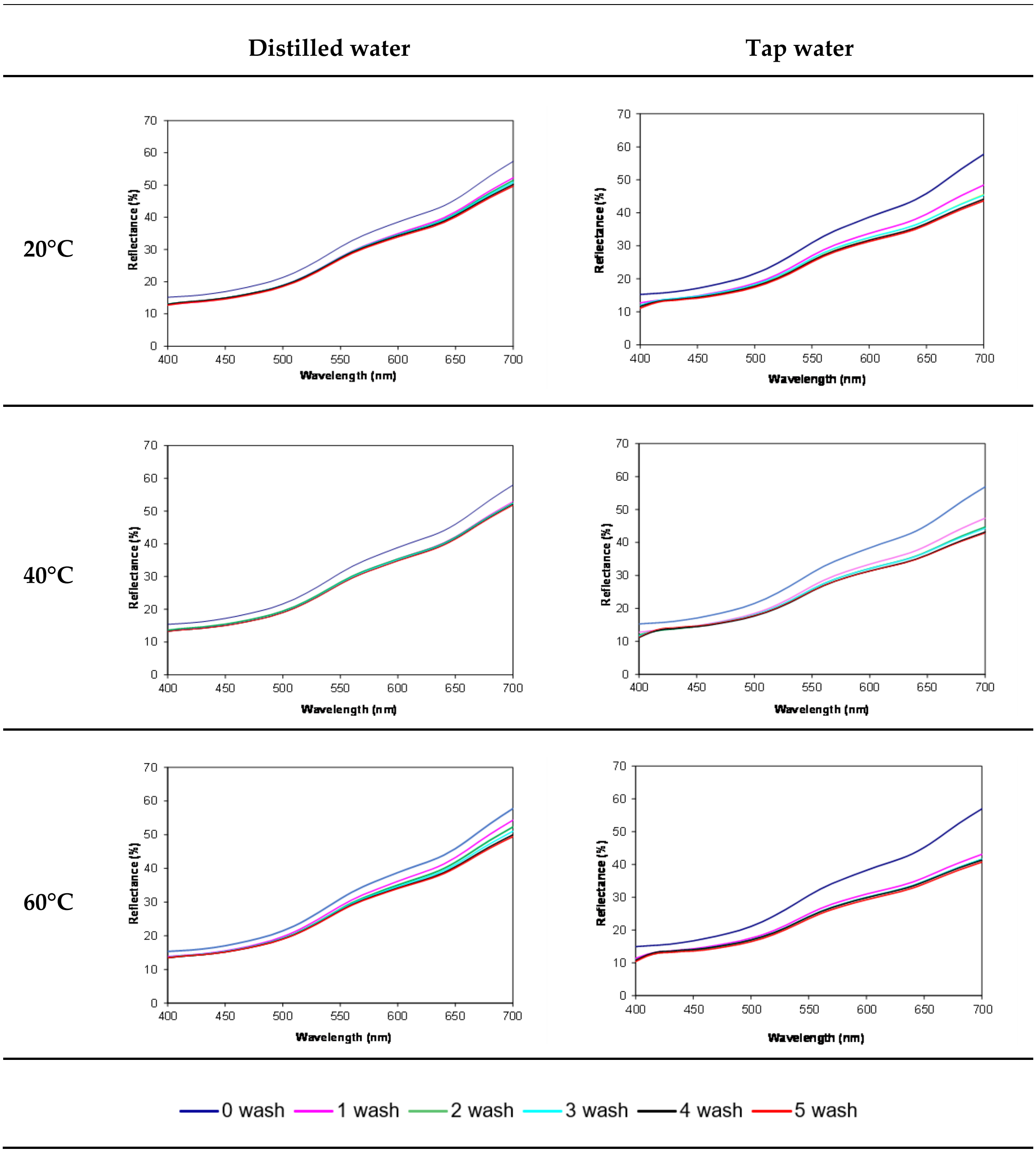

Figure S2. Reflectance curves of the substrates washed with detergent A.

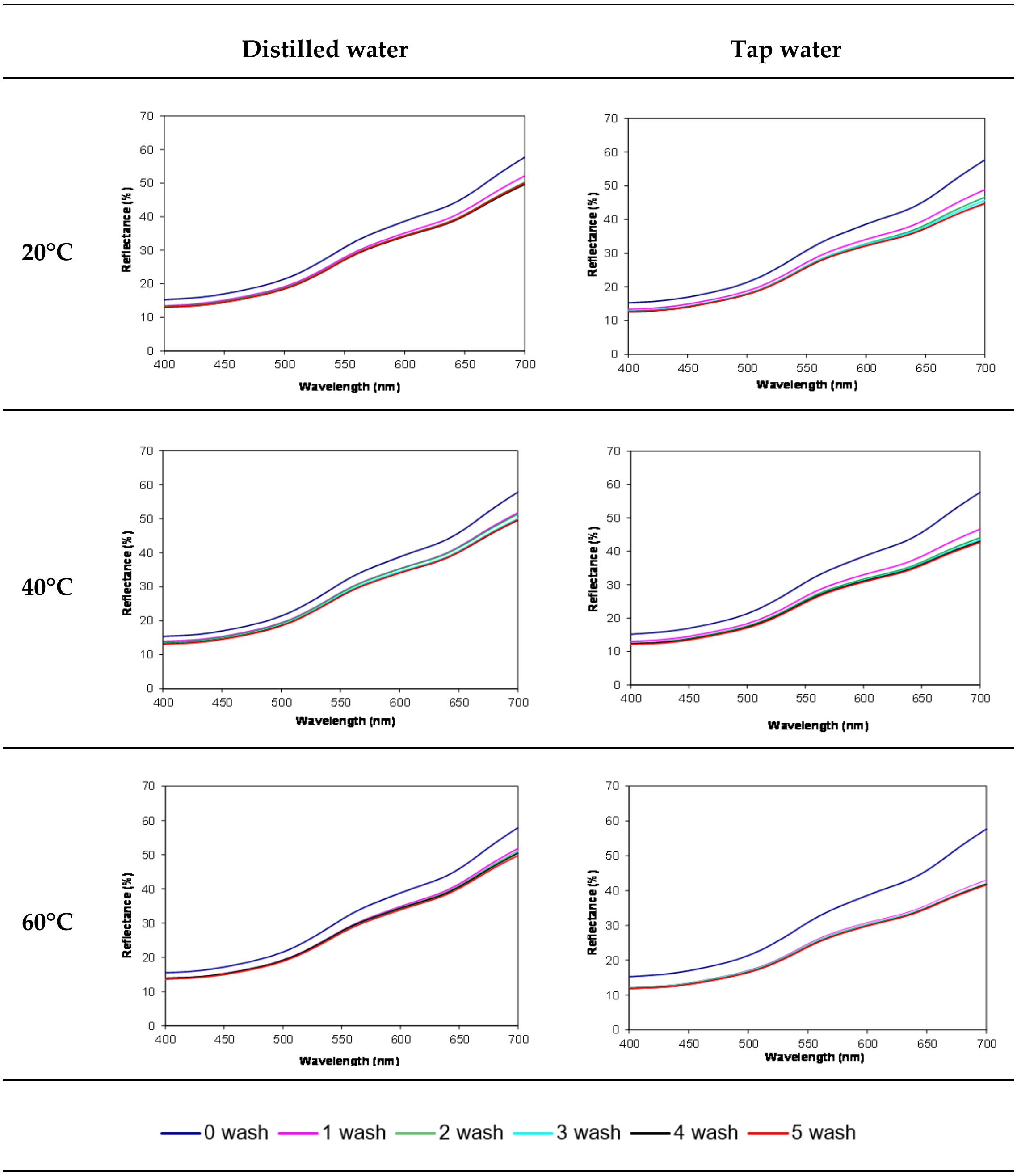

Figure S3. Reflectance curves of the substrates washed with detergent B.

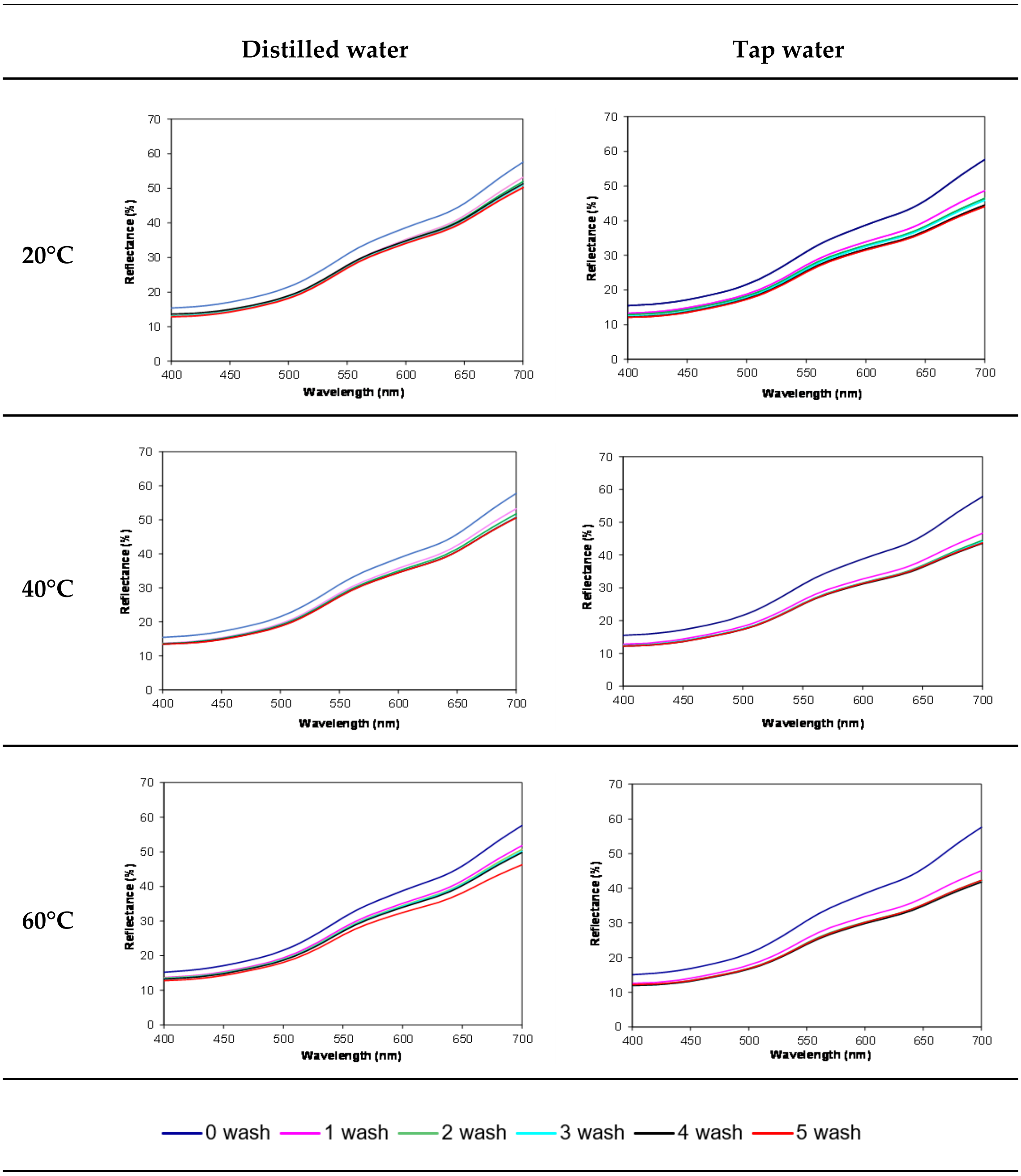

Figure S4. Reflectance curves of the substrates washed with detergent C.

Table S1. CIE*L*a*b** coordinate values before and after washing with detergent A under different conditions.

Type of water	T (°C)	Wash number	*L**	*a**	*b**	C^*_{ab}	h_{ab}	ΔE^*_{ab}
Distilled	20	0	62.15	9.23	23.14	24.92	68.25	
		1	59.46	9.35	23.33	25.14	68.16	2.70
		2	59.28	9.23	22.77	24.57	67.94	2.89
		3	59.27	9.11	22.71	24.47	68.15	2.92
		4	59.11	9.02	22.42	24.17	68.08	3.14
		5	58.80	9.04	22.60	24.34	68.20	3.40
	40	0	62.43	9.22	23.04	24.82	68.20	
		1	59.94	9.16	22.88	24.64	68.17	2.49
		2	59.94	9.08	22.62	24.38	68.12	2.52
		3	59.67	9.22	23.09	24.86	68.23	2.75
		4	59.46	9.25	22.81	24.62	67.93	2.98
		5	59.40	9.21	22.83	24.62	68.04	3.04
	60	0	62.31	9.29	23.20	24.99	68.18	
		1	60.44	9.30	23.28	25.06	68.23	1.87
		2	59.68	9.17	22.70	24.48	68.01	2.68
		3	59.49	8.90	22.16	23.88	68.11	3.03
		4	59.18	8.78	22.06	23.74	68.29	3.37
		5	59.08	8.69	21.82	23.49	68.29	3.56
Tap	20	0	62.31	9.21	23.01	24.78	68.19	
		1	58.75	8.81	22.12	23.80	68.29	3.69
		2	57.81	8.55	21.43	23.07	68.26	4.82
		3	57.96	8.44	21.03	22.66	68.12	4.84
		4	57.33	8.46	20.86	22.51	67.93	5.48
		5	56.97	8.53	20.81	22.49	67.73	5.82
	40	0	62.17	9.01	22.90	24.61	68.52	
		1	58.52	8.73	22.00	23.67	68.37	3.77
		2	57.65	8.44	21.07	22.70	68.18	4.91
		3	57.68	8.37	20.75	22.38	68.03	5.02
		4	57.12	8.37	20.28	21.94	67.58	5.72
		5	57.15	8.23	19.76	21.41	67.40	5.98
	60	0	61.93	9.22	23.24	25.00	68.36	
		1	56.82	8.34	20.22	21.88	67.58	6.00
		2	55.99	8.37	19.85	21.54	67.15	6.89
		3	56.05	8.35	19.51	21.22	66.83	7.02
		4	55.97	8.34	19.35	21.07	66.69	7.18
		5	55.39	8.48	19.51	21.27	66.52	7.57

Table S2. CIE$L^*a^*b^*$ coordinate values before and after washing with detergent B under different conditions.

Type of water	T (°C)	Wash number	L^*	a^*	b^*	C^*_{ab}	h_{ab}	ΔE^*_{ab}
Distilled	20	0	62.24	9.30	23.17	24.97	68.14	
		1	59.66	9.26	22.97	24.77	68.05	2.58
		2	59.18	8.99	22.81	24.52	68.48	3.09
		3	58.86	9.08	23.02	24.75	68.47	3.39
		4	59.14	8.96	22.85	24.54	68.60	3.13
		5	58.99	9.13	23.10	24.84	68.43	3.25
	40	0	62.29	9.28	23.28	25.06	68.26	
		1	59.87	8.97	22.75	24.45	68.48	2.49
		2	59.73	8.99	23.09	24.78	68.73	2.58
		3	59.14	8.93	23.20	24.86	68.94	3.17
		4	58.91	8.89	22.84	24.51	68.74	3.43
		5	58.86	8.97	23.04	24.72	68.73	3.45
	60	0	62.39	9.23	23.07	24.85	68.19	
		1	59.56	9.07	22.57	24.32	68.10	2.87
		2	59.39	8.90	22.41	24.11	68.33	3.08
		3	59.17	8.85	22.40	24.09	68.44	3.31
		4	59.33	8.72	22.16	23.82	68.52	3.23
		5	58.91	8.82	22.08	23.77	68.21	3.63
Tap	20	0	62.20	9.28	23.18	24.97	68.19	
		1	59.00	8.80	22.41	24.08	68.55	3.33
		2	58.00	8.73	22.45	24.08	68.74	4.31
		3	57.99	8.49	22.03	23.61	68.94	4.43
		4	57.58	8.46	22.03	23.60	68.99	4.83
		5	57.67	8.42	22.08	23.63	69.13	4.74
	40	0	62.11	9.31	23.06	24.87	68.01	
		1	58.27	8.51	21.88	23.48	68.75	4.09
		2	57.27	8.30	21.69	23.22	69.06	5.13
		3	56.89	8.40	21.73	23.30	68.86	5.47
		4	56.81	8.26	21.56	23.09	69.03	5.61
		5	56.53	8.34	21.62	23.17	68.91	5.84
	60	0	62.22	9.33	23.14	24.95	68.03	
		1	56.55	8.33	21.59	23.14	68.89	5.96
		2	56.29	8.22	21.13	22.67	68.75	6.36
		3	56.12	8.33	21.20	22.78	68.55	6.48
		4	55.81	8.42	21.22	22.83	68.35	6.75
		5	55.71	8.46	21.15	22.78	68.19	6.85

Table S3. CIE*L*a*b** coordinate values before and after washing with detergent C under different conditions.

Type of water	T (°C)	Wash number	*L**	*a**	*b**	C^*_{ab}	h_{ab}	ΔE^*_{ab}
Distilled	20	0	62.28	9.16	23.03	24.79	68.32	
		1	59.51	9.60	23.87	25.73	68.10	2.93
		2	59.30	9.44	23.37	25.21	68.01	3.01
		3	59.01	9.32	23.19	24.99	68.10	3.28
		4	59.44	9.16	22.81	24.58	68.12	2.85
		5	58.75	9.34	23.38	25.17	68.22	3.55
	40	0	62.33	9.22	22.94	24.73	68.10	
		1	60.10	9.25	23.15	24.93	68.22	2.24
		2	59.59	9.16	22.80	24.57	68.10	2.74
		3	59.23	9.10	22.71	24.46	68.17	3.11
		4	59.19	9.14	22.70	24.48	68.07	3.14
		5	59.15	9.17	22.76	24.54	68.06	3.19
	60	0	62.32	9.24	23.08	24.86	68.18	
		1	59.75	9.03	22.61	24.35	68.23	2.62
		2	59.27	9.03	22.31	24.07	67.96	3.15
		3	59.09	9.07	22.39	24.15	67.96	3.31
		4	58.87	9.03	22.35	24.11	67.99	3.53
		5	57.84	8.62	21.76	23.40	68.38	4.72
Tap	20	0	62.36	9.16	22.97	24.73	68.26	
		1	58.92	8.69	22.32	23.95	68.73	3.54
		2	58.20	8.46	22.12	23.69	69.06	4.31
		3	57.76	8.59	22.68	24.26	69.25	4.65
		4	57.26	8.44	22.30	23.84	69.28	5.20
		5	57.00	8.51	22.26	23.83	69.09	5.45
	40	0	62.38	9.23	23.01	24.79	68.14	
		1	58.06	8.45	21.95	23.52	68.95	4.52
		2	57.13	8.43	21.69	23.27	68.75	5.47
		3	57.05	8.37	21.63	23.20	68.84	5.57
		4	56.81	8.36	21.73	23.28	68.96	5.78
		5	56.95	8.42	21.85	23.41	68.93	5.62
	60	0	62.12	9.36	23.25	25.06	68.06	
		1	57.41	8.31	21.66	23.20	69.01	5.07
		2	56.13	8.31	21.14	22.71	68.55	6.43
		3	55.89	8.29	21.08	22.66	68.54	6.68
		4	55.78	8.33	20.98	22.58	68.35	6.81
		5	56.03	8.40	20.97	22.59	68.17	6.57
